# Capturing Peptide–GPCR Interactions and Their Dynamics

**DOI:** 10.3390/molecules25204724

**Published:** 2020-10-15

**Authors:** Anette Kaiser, Irene Coin

**Affiliations:** Faculty of Life Sciences, Institute of Biochemistry, Leipzig University, Brüderstr. 34, D-04103 Leipzig, Germany; irene.coin@uni-leipzig.de

**Keywords:** GPCR activation, peptide–GPCR interactions, structural dynamics of GPCRs, peptide ligands, crosslinking, NMR, EPR

## Abstract

Many biological functions of peptides are mediated through G protein-coupled receptors (GPCRs). Upon ligand binding, GPCRs undergo conformational changes that facilitate the binding and activation of multiple effectors. GPCRs regulate nearly all physiological processes and are a favorite pharmacological target. In particular, drugs are sought after that elicit the recruitment of selected effectors only (biased ligands). Understanding how ligands bind to GPCRs and which conformational changes they induce is a fundamental step toward the development of more efficient and specific drugs. Moreover, it is emerging that the dynamic of the ligand–receptor interaction contributes to the specificity of both ligand recognition and effector recruitment, an aspect that is missing in structural snapshots from crystallography. We describe here biochemical and biophysical techniques to address ligand–receptor interactions in their structural and dynamic aspects, which include mutagenesis, crosslinking, spectroscopic techniques, and mass-spectrometry profiling. With a main focus on peptide receptors, we present methods to unveil the ligand–receptor contact interface and methods that address conformational changes both in the ligand and the GPCR. The presented studies highlight a wide structural heterogeneity among peptide receptors, reveal distinct structural changes occurring during ligand binding and a surprisingly high dynamics of the ligand–GPCR complexes.

## 1. Introduction

More than a hundred G protein-coupled receptors (GPCRs) in the human body are activated by endogenous peptide or protein ligands [1]. This is the case of all GPCRs of the secretin family, and branch β of the rhodopsin family, among many others [2]. Peptide and protein GPCRs are involved in many physiological processes and are the main molecular pharmacological targets. A total of 30% of currently marketed drugs target GPCRs [3,4]. Although peptide and protein receptors are still underrepresented in the clinical intervention, the number of peptide therapeutics is constantly increasing [5,6]. Understanding how peptide ligands interact to their receptors is an essential step toward the development of more potent and selective drugs.

Traditionally, peptide–receptor interactions have been investigated with indirect methods based on mutagenesis (structure–activity relationship studies, SAR). Recent improvements of crystallographic techniques and of techniques of cryo-electron microscopy (cryo-EM) have allowed achieving 3D information about GPCRs with atomic resolution. Along with a large number of structures of GPCR complexes bound to a small-molecule ligand, a group of structures of GPCRs bound to peptide ligands have been solved, although these are not always the endogenous ligands. These include, in the rhodopsin branch, neurotensin at the NTS_1_R [7,8,9,10], endothelin 1 and 3 at the ET_B_ receptor [11,12], angiotensin at the AT_1_R [13,14], as well as synthetic endorphin-derivatives at the μ and δ-opioid receptors [15,16], and an apelin-mimetic at the APJR [17]. In the secretin family, a number of G protein bound peptide–receptor complexes have been resolved via cryo-EM, which includes glucagon- and glucagon-like peptide 1 (GLP-1) analogues at the glucagon receptor [18,19] and GLP_1_R [20,21,22], respectively; urocortin (Ucn) and corticopin-releasing factor (CRF) at CRF_1_R and CRF_2_R [23,24], pituitary adenylate cyclase-activating peptide (PACAP) at PAC_1_R [24], parathyroid hormone at the PTH_1_R [25], and calcitonin-derivatives at the CGRP receptor [26,27,28].

**Figure 1:**
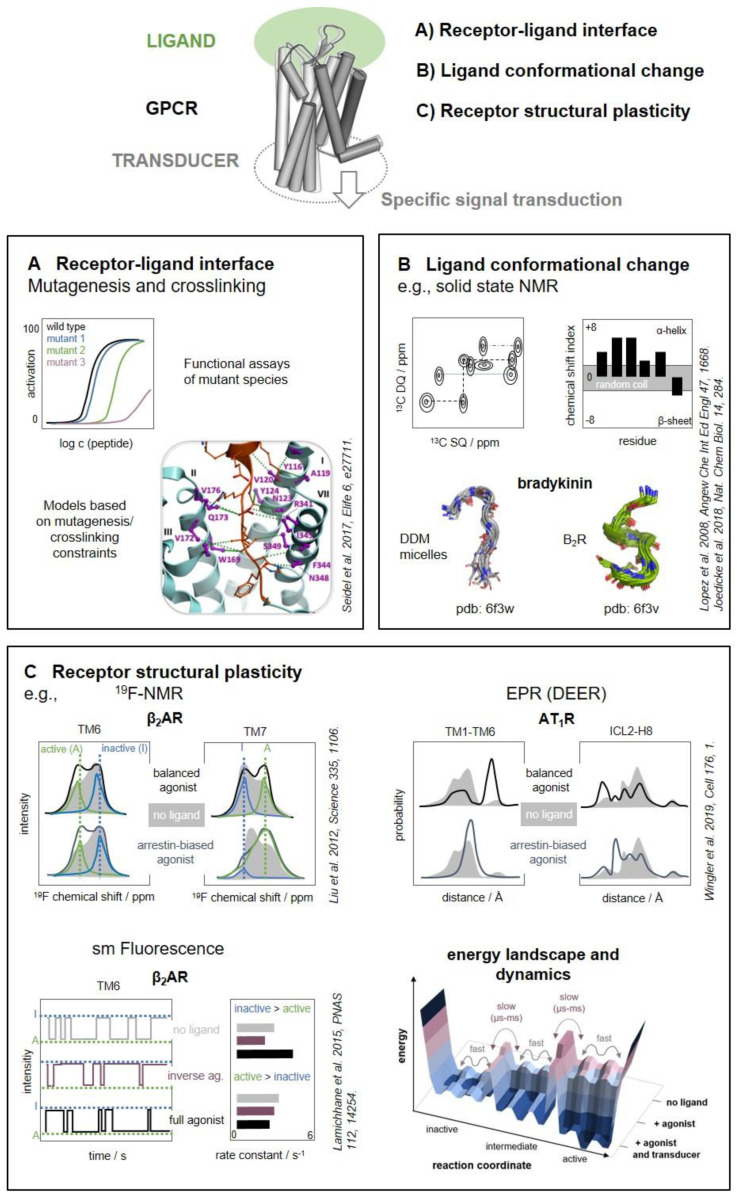
Methods to study peptide–receptor interactions and the dynamic ligand–receptor complex. It is essential to define the receptor–ligand interface (**A**), the conformational changes of the ligand during binding (**B**), and the structural plasticity of the receptor itself (**C**) to understand the specific signal transduction. (**A**) Structure–activity relationship (SAR) studies reveal key residues, both of the ligand and of the receptor, that are crucial for the interaction. Photo- and chemical crosslinking experiments reveal points of proximity between ligand and receptor, which guide the building of molecular models of the peptide–receptor complex. (**B**) The conformation of the receptor-bound ligand can be determined by solution or solid-state NMR, which typically shows order-to-disorder transformation or vice versa. The conformational transitions may reflect an induced fit or conformational selection mechanism. (**C**) NMR, electron paramagnetic resonance (EPR), and fluorescence techniques with residue-specific labels have been applied to illuminate the structural plasticity of the receptor. Different agonists specifically modulate the conformations, for instance G protein-biased versus arrestin-biased ligands. NMR shows changes in the chemical environment and dynamics (exchange rates) of states. Pulsed EPR (DEER) displays the equilibrium of receptor conformations based on the distances between two spin labels. Single-molecule fluorescence spectroscopy directly shows the transitions of a single receptor between different inactive/active states over time. Collectively, these studies suggest a general scheme for the energy landscape of most GPCRs. Inactive, intermediate, and active states (reaction coordinate, *x*-axis) are not a single states, but contain multiple energetically similar conformations (energy, *y*-axis) which interchange rapidly. In contrast, the energy barriers along the reaction coordinate are high, leading to a slow (µs-ms) conversion between inactive and active states. The active states are only energetically favored in the presence of agonist and transducer.

These studies have greatly increased our structural and functional understanding of these receptors [29,30,31,32], and also identified some common structural hallmarks in each receptor family. For instance, there is a short β-hairpin located in the second extracellular loop (ECL2) of rhodopsin-like peptide GPCRs [29], which facilitates access to the transmembrane binding cavity. The orientation and binding depth of peptide ligands in the transmembrane binding pocket is variable, and peptides have in general a larger contact interface with the extracellular loops compared to small-molecule ligands. Secretin-like GPCRs display a generally more open, V-shaped transmembrane binding pocket compared to rhodopsin-like GPCRs. They also feature a large N-terminal extracellular domain (ECD) that importantly contributes to peptide binding [24,33,34]. Strikingly, binding modes of peptide ligands are variable even among receptors of the same phylogenetic family and are hardly generalizable, which reflects the high specificity of their physiological function. Moreover, GPCRs are intrinsically highly flexible molecules, and the sheer size of the ligands adds even more flexibility and variability to the system. It is becoming increasingly evident that protein dynamics are of critical importance for receptor function [35,36,37]. This applies to the recognition of the ligands, but also for the recognition of the signaling transducers (G proteins and arrestins) to a given GPCR, which can be investigated by the same set of methods. How GPCRs select specific transducers remains enigmatic and cannot be firmly predicted from sequence or structural data alone [38,39,40]. Instead, the dynamic of the interactions and the existence several intermediate states may drive recognition and activation of the effector [19,41,42]. Thus, in order to understand the specific activation and signal transduction of GPCRs, it is essential to define the receptor–ligand interface (Figure 1A), the conformational changes of the ligand during binding (Figure 1B), and the structural plasticity of the receptor itself (Figure 1C).

Here, we describe biochemical and biophysical techniques to study peptide–receptor interactions and the dynamic ligand–receptor complex (Figure 1). Many of these techniques contain structural and dynamic information, and can in principle be applied to the ligand–receptor interface as well as the receptor–transducer interface. We cover mutagenesis and crosslinking, as well as spectroscopic techniques: nuclear magnetic resonance (NMR), electron paramagnetic resonance (EPR), fluorescence, and Fourier-transform infrared spectroscopy (FTIR). We include hydrogen/deuterium exchange (HDX) and hydroxyl radical-footprinting (HRF) coupled with mass spectrometry (MS). It is important to remark that there is no single, ‘perfect’ technique to address all questions about peptide–ligand interactions and their dynamics. Each approach has its own structural and temporal resolution and technical limitations, which we will discuss as well. In fact, most methods give complementary information. Ideally, several of these methods should be combined with high-resolution structural information to enable a comprehensive view of the functioning of these systems at the molecular level. Complementary data can be used as part of an integrated structural biology approach [43,44,45,46] to feed information into homology modeling or compare with molecular dynamics simulations.

We will first discuss strategies to define the ligand–receptor contact interface; second, methods to delineate structural transitions of the peptide/protein ligand. In a third part, we will discuss techniques to illuminate structural and dynamic transitions within the GPCR itself.

## 2. The Contact Interface

Crystallography or cryo-EM can characterize the interaction interface between peptide ligands and their GPCRs with high precision. However, only receptors that can be produced in large amounts and purified in a functional form can be investigated in this way. In fact, obtaining crystals and EM images of any GPCR complex is still a long and cumbersome endeavor, with no guarantee of success. Although about two dozen structures of peptide GPCRs have been solved, only few of these structures represent the receptor in complex with the natural ligand/agonist, whereas most of them depict complexes with small molecules [1,47]. In any case, such structural techniques lack the dynamic aspect of the interaction. Moreover, such procedures reconstitute the receptors in an artificial environment, which might affect the natural interaction. In this context, classical biochemical methods such as mutagenesis and crosslinking are still of utmost importance both to investigate peptide–receptor complexes that elude the direct structural characterization and to address interactions in the environment of the live cell (Figure 1A). It is important to remark that crystal structures can hardly predict the effect of a mutation on the activity of a peptide ligand. Still nowadays structure–activity relationship (SAR) studies are indispensable for the detailed characterization of a peptide–GPCR interaction and for the fine tuning of the activity of ligands developed for therapeutic purposes. 

Mutagenesis studies, i.e., structure–activity relationship studies, are based on the concept that a mutation introduced either into the ligand or into the receptor perturbs the ligand–receptor interaction when it is located along the interaction interface. This is reflected by changes in biological activity observed in binding or activation assays, and reveals key residues both in the ligand and in the receptors. A common procedure for the ligand’s perspective is the systematic substitution of each residue of the peptide with an Ala, which carries a neutral side chain. Among the first studies of this kind, back in early 1990s, it is worth mentioning the Ala-scan of the 40-mer CRF [48] and of NPY [49]. These studies are usually meant to reveal whether a position is “tolerant” toward mutation or not. The method cannot distinguish whether an observed effect is caused by the disruption of a specific interaction involving the side chain of the mutated amino acid or by a change of its general character (hydrophilic/hydrophobic, charged, aromatic). This information can be acquired using the “slight alteration” method (also known as minimal replacement or conservative mutagenesis) [50]. The idea is to substitute one residue with residues carrying similar side chains. For instance, a Phe residue of the neuropeptide CRF that could not be substituted with Ala could be well substituted with Trp, yielding an even more potent agonist [50]. Such a result reveals that key for the interaction is not a specific interaction between the receptor and the side chain of Phe itself, but the presence of a bulky hydrophobic amino acid. Other approaches are D-amino acid substitutions [51], which reveal the importance of a specific side chains without changing the physicochemical properties of the ligand, or double-D substitution [52] and proline scanning [53], which address structural aspects of the peptide. Another mutagenesis approach is the swapping of Lys/Glu and in general polar residues between ligand and receptor, which reveals the contribution of salt bridges and polar interactions in the ligand–receptor interface (complementary mutagenesis). For instance, a ligand may lose its activity when a Lys/Arg residue is mutated to Glu. If the activity is recovered when a Glu/Asp residue in the receptor is mutated to Lys/Arg, an intermolecular ionic interaction between the two positions can well be hypothesized [54]. However, these drastic alterations of the local environment of the receptor and peptide often provoke artificial conformational changes and render the receptor and/or ligand inactive. This issue is circumvented by double cycle mutagenesis [55]. The method reveals intermolecular pairs of interacting residues in the ligand–receptor interface. The underlying assumption is that a mutant receptor showing decreased activity when tested with the wild type ligand (first cycle, mutagenesis at the receptor), will feature the same activity if the ligand is mutated at the exact position that interacts with the mutated position of the receptor (second cycle, mutagenesis of the ligand combined with the receptor mutants). In other words, if one partner of a peptide–receptor interaction has already been removed (e.g., in the mutant receptor) the mutation of the counterpart in the peptide will show no further effects. In contrast, if the peptide is mutated in a position that belongs to another site of interaction, additional effects will be observed. This strategy has allowed the identification of ligand–receptor salt-bridges of conserved residues in the NPY system by using Ala mutants [56]. This concept can also be applied to identify non-polar interactions by mutating interacting Leu/Ile residues to the smaller Ala or even polar Asn/Gln [57,58]. One caveat of the double-cycle mutagenesis is that it works best for relatively isolated interactions. In complex binding pockets with multiple interactions of each residue, often some further shift of potency occurs when mutating the interacting position in the ligand, which complicates the analysis and requires double/triple mutants on the receptor side. Ligand–receptor contacts derived from double-cycle mutagenesis or swapping of salt bridges can be used to generate structural models of the peptide–receptor complexes. Even two or three direct interactions are enough to guide the docking process and allow the construction of high-quality models that satisfy all available biological data (as described in [57,58,59]). 

These concepts apply to investigations carried out both from the side of the ligand and from the side of the receptor. To investigate the function of larger receptor domains, receptor chimeras are widely applied. In this case whole domains of a GPCR (for instance a loop, or the C-terminus) are substituted for the same elements taken from another receptor and the functional effect is analyzed. The approach has been widely applied, for instance, to dissect the peptide binding mode at secretin-like GPCRs (reviewed in [60]). This approach works best for entire domains and in the N- and C-termini. Exchanging loop regions is more difficult as the flanking positions need to be chosen carefully to avoid affecting receptor folding. 

While it would be impossible to review the huge number of example of mutagenesis studies and studies with receptor chimeras carried out at peptide GPCRs, we highlight some recent works that applied large scale Ala mutagenesis to systematically scan either the whole extracellular domains of a GPCR or even the whole receptor. These works involve the generation and the functional characterization of hundreds of Ala-GPCR mutants, which may appear as a “brute force” approach. However, the high efficiency of modern molecular biology tools, as well as the availability of high-throughput mutagenesis protocols and software for primer design [61] make large scale mutagenesis smoothly accessible. Importantly, high throughput assays must be available to investigate the effect of the mutation on the different signaling pathways triggered by the GPCR. The approach is overall very powerful. For instance, systematic Ala scan of the extracellular juxtamembrane domain of the GLP_1_R has provided molecular insights into the mechanism of receptor activation and spotted elements modulating biased signaling [62]. In a smaller scale, mutation of polar residues of the same receptor to Ala or other amino acids has identified a polar network, conserved among secretin-like GPCRs, that contributes to its structural stability and controls signal transmission and specificity [63,64,65]. Comprehensive mutagenesis at the CXCR4 has revealed 41 amino acids that are critically required for receptor activation by the chemokine ligand CXCL12 (stromal cell-derived factor 1) [66].

### Crosslinking

Mutagenesis studies are an indirect method to characterize ligand–receptor interactions and have their limitations. For instance, if a certain mutation has detrimental effect on the activity of a ligand or on the function of a receptor, mutagenesis cannot tell whether this effect is due to the disruption of a crucial interaction or whether the mutation is rather affecting the folding of the mutated species. A direct biochemical method to identify contact points between ligand and receptor is crosslinking. There are two types of crosslinking: photo-crosslinking and chemical crosslinking (or pair-wise crosslinking).

**Figure 2:**
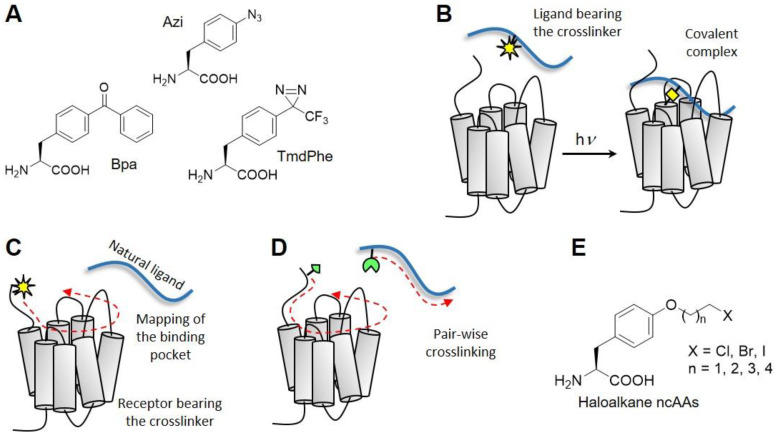
Photo- and chemical crosslinking to investigate peptide–receptor interactions. (**A**) Examples of amino acids for photo-crosslinking: *p*-benzoyl-Phe (Bpa); *p*-azido-Phe (Azi); 4-(3-trifluoromethyl)-3H-diazirine-3-yl-Phe ((Tmd)Phe). (**B**) Photo-crosslinking using crosslinkers chemically incorporated into the peptide. (**C**) Photo-crosslinking mapping using crosslinkers genetically incorporated into the receptor. (**D**) the concept of pair-wise crosslinking (or 2D crosslinking): the reaction between the two moieties takes places only when they come into reciprocal proximity. (**E**) Genetically encoded halo-alkane non-canonical amino acids (ncAAs) for pair-wise crosslinking.

Photo-crosslinking is based on the use of chemical moieties, usually benzophenone, azide, or diazirine, that are inert under physiological conditions and turn into very reactive radical species when activated by ultraviolet light [67] (Figure 2A). These species insert into bonds of other molecules coming in their proximity, thus generating a covalent complex. In classical photo-crosslinking experiments (photo-affinity labeling, Figure 2B), a photo-activatable moiety is incorporated into the peptide ligand by chemical synthesis. The ligand is applied to cells expressing the desired receptor and photo-crosslinking activated by irradiation. To identify the region of the receptor captured by the photo-label, the ligands are equipped with a radioactive moiety. The crosslinked complex is fragmented using residue-specific chemical reagents (BNPS-skatole, cuts after Met) or amino acid specific enzymes (Lys-C, Glu-C), and the molecular weight of the fragment containing the radiolabel is estimated by SDS-PAGE analysis. By combining the results from different cleavage procedures, the region of crosslinking and sometimes even the crosslinking site can be inferred. This information provides intermolecular spatial constraints for building molecular models of peptide–receptor complexes. First photo-activatable peptides contained *p*-azido-phenylalanine (Azi) residues and have been developed in the late 70s [68]. Due to the low stability of Azi both toward light and standard conditions of peptide synthesis, Azi-peptides have to be synthesized as amino-precursors, which are converted to the photoactive species shortly before the experiment. The use of Azi in peptide ligands has been later replaced by the use of *p*-benzoyl-phenylalanine (Bpa). Bpa is stable during peptide synthesis and under the normal light conditions of a laboratory. It is activated by biocompatible UV light of longer wavelength (365 nm) and offers a better photochemistry. First models of ligand–bound secretin-like GPCRs were all built on the basis of constraints from photo-affinity labeling using Bpa-ligands [69,70,71]. Nowadays, it is sometimes possible to identify the residue captured in the crosslinking reaction using tandem mass spectrometry (MS/MS). However, MS/MS on GPCRs is only possible when large amounts of isolated receptor are available, as it is for instance the case of rhodopsin [72] or receptors obtained by either yeast [73,74,75] or bacterial expression [58]. 

Modern methods of unnatural amino acid mutagenesis have enabled the genetic incorporation of photo-crosslinking moieties into proteins as they are assembled by the ribosomal machinery. The technique is based on the reassignment of an amber stop codon to a non-canonical amino acid (ncAA), which carries the crosslinker on the side chain. Demonstrated in live cell at the beginning of the 2000s, the expanded genetic code technology can be nowadays applied almost on a routine basis also in non-specialized laboratories [76,77]. A number of amino acids bearing crosslinking moieties applicable to the study of protein interactions, including Azi and Bpa, have been genetically encoded (reviewed in [78]). This has opened up to perform photo-affinity crosslinking experiments with the crosslinker installed into the receptor rather than into the ligand [78,79]. To determine ligand binding sites, an ncAA bearing in the side chain a photo-activatable moiety (usually Azi or Bpa) is systematically incorporated throughout the juxtamembrane domain of the GPCR using a mammalian cell host (usually HEK293 or 293T cells) (Figure 2C). The ligand is applied to the live cells and crosslinking triggered with biocompatible UV light (365 nm). If the bound ligand lies within the radius of reach of the crosslinker (e.g., ~9 Å from the Cβ of Azi), it gets captured by the photo-active moiety. A covalent ligand–receptor complex is formed, which is detected in Western blot at the approximate molecular weight of the receptor using an anti-ligand antibody. In this way, the footprint of the ligand on the receptor is determined, which unveils the location and the shape of the ligand binding pocket. 

Photo-crosslinking mapping using genetically encoded crosslinkers was first demonstrated in the early 2010s, as it revealed the binding site of the 16-mer peptide T140 on the CXC chemokine receptor 4 [80] and the binding pocket of the neuropeptide urocortin1 (Ucn1) on the corticotropin releasing factor receptor type 1 (CRF_1_R) [81,82]. Since then, the approach has been employed to determine the binding site of allosteric drugs on the chemokine receptor 5 [83], the binding mode of substance P on the neurokinin-1 receptor [84], the binding site of exendin-4 on the glucagon-like peptide-1 (GLP-1) receptor [85], and the binding mode of the calcitonin gene-related peptide (CGRP) to the calcitonin receptor-like receptor (CLR) [86]. The approach offers single-residue resolution, which is precise enough to distinguish binding modes of pharmacologically distinct ligands to the same receptor [87]. Genetically encoded photo-crosslinkers have also revealed high and low affinity binding sites of antidepressant drugs on the human serotonin transporter [88,89], as well as details of the insulin–insulin receptor complex [90]. 

As powerful as this method is, it fails to reveal which residue of the ligand is captured by the crosslinker incorporated into the receptor. This information is usually critical to derive position and orientation of peptide ligands in their binding pocket. Peptides are highly flexible and can assume different conformations, so that unrestrained docking of large peptide ligands into the receptor binding pocket may not be reliable enough to explore details of the real interaction. To derive this information, photo-crosslinking mapping can be fruitfully combined with chemical crosslinking. Chemical crosslinking, or pair-wise crosslinking (Figure 2D), relies on pairs of chemical moieties that react selectively with each other when they come into reciprocal proximity. One moiety is installed into the ligand and the other into the receptor. The occurring of the chemical reaction reveals intermolecular pairs of proximal positions, which can be translated into spatial constraints for the construction of accurate models of ligand–receptor complexes, similarly as outlined above for ligand–receptor contacts derived from double cycle mutagenesis. 

Disulfide trapping is a very common procedure to perform pair-wise crosslinking and has been extensively applied to study peptide–receptor interactions [91,92]. In this case, the pair of mutually reactive amino acids consists of two Cys residues. Sets of Cys-ligand analogues are combined with sets of Cys-receptor mutants (2D crosslinking) and the occurring of the reaction is assessed via SDS-PAGE performed in the absence of reducing agents. Systems investigated via disulfide trapping include ligand-bound class B GPCRs [93,94,95], chemokine receptors [96,97], the M3 muscarinic acetylcholine receptor [98,99,100], and other receptors [101,102]. Disulfide trapping has been used also to study GPCR dimerization [103,104,105,106] and to characterize interaction interfaces between GPCRs and G protein or arrestin [107]. Unfortunately, disulfide trapping does not always yield clear results, probably because of the requirement of nonreducing conditions during analysis of samples [93,95,96,98]. 

Another way to derive intermolecular pairs of proximal amino acids involve the use of non-canonical amino acids carrying mildly electrophilic moieties that attack nucleophiles present in canonical amino acids to irreversibly form a covalent bond. Several electrophiles which selectively react with Cys [108,109,110] or other nucleophilic amino acids [111] have been genetically encoded (reviewed in [78]). Importantly, the electrophilic moiety is stable under physiological conditions and reacts with the target nucleophile only when the two groups come into reciprocal proximity (proximity-enhanced reactivity) [108,112,113]. The choice of the electrophile and nucleophile can vary, but based on our experience the use of halo-alkanes combined to Cys is very reliable for investigating GPCR interactions [114]. The electrophile can be incorporated either genetically into the receptor or chemically into the ligand. The second approach may be preferable, as sets of Cys-receptors are usually expressed with higher and more homogeneous yields compared to sets of receptors containing ncAAs. Combining chemical crosslinking with a preliminary mapping of the receptor surface via photocrosslinking greatly reduces the size combinatorial matrix, as only receptor positions belonging to the binding pocket (the ligand footprint) need to be tested.

Using this two-steps method, we have discovered in 2013 how Ucn1 binds to the CRF_1_R [82]. We have built a molecular model for the Ucn1-CRF_1_R supported by about 50 experimental constraints, which is the most detailed ever published for a peptide in complex with a GPCR (Figure 1A). By investigating binding of peptide agonists and antagonists on the CRF_1_R, we have shown that bound antagonists adopt a different conformation with respect to the agonists [87]. Importantly, these models have revealed information not only about the conformation of the ligands, but also about that of the receptor. For instance, we have shown that the tilted conformation of helix VII observed in the crystal structure of the CRF_1_R in complex with the allosteric antagonist CP-376395 [115] is not compatible with the peptide-bound receptor. Moreover, by comparing models of agonist- and antagonist-bound CRF_1_R, we have gained important hints on the mechanisms of receptor activation. Finally, by exploring the models with molecular dynamics simulations, we have identified major regions of flexibility in the complex, validated on the basis of photo-crosslinking results. At the time these models were built, the structure of the peptide-bound full length CRF_1_R was still elusive. Gratifyingly, these models are very well compatible with the very recently solved structures of CRF- and Ucn-CRF_1_R complexes [23,24], which demonstrates the power of the approach and the predictive value of the models. Indeed, in the lack of an atomic structure, computational models based on existing crystal structures of homolog receptors and guided by spatial constraints derived via crosslinking represent the best possible approximation of a peptide–GPCR complex [116]. Lately, we have shown that the same approach can be applied to investigate interactions of GPCRs with intracellular effectors, such as arrestins [117]. 

It is important to remark that photo- and biochemical crosslinking provide information about the ligand–receptor interaction directly from the environment of the live cell, which is not accessible to crystallography and cryo-EM. Moreover, crosslinking can capture transient states that are not accessible to structural snapshots of energetically stable complexes, thus providing further information about binding dynamics, as it is for instance the case of the very recent structure of the G protein-bound secretin-secretin receptor complex [118].

## 3. A Ligand’s Perspective

Many peptidic GPCR ligands display a regular α-helical structure in solution, as observed for basically all ligands of the secreting family [60], and also some ligands of rhodopsin-like receptors, for instance neuropeptide Y (NPY) [119,120]. Moreover, small protein ligands like chemokines also display well-defined folds (reviewed in [121]). Conversely, there are several peptide ligands that lack a defined secondary structure in solution, like for instance neurotensin (13 aa, [122,123]) or ghrelin (28 aa, [124,125,126]). In the current paradigm, ligand–receptor recognition and functional versatility is mediated by both structured and disordered regions [37,127,128,129]. Ligand binding to GPCRs involves some structural change at both the ligand and the receptor (Figure 1B). Based on the analysis of available structures, ligands of the secretin family keep their helical conformation in the receptor-bound state. Only the very N-terminal segment, which interacts with the transmembrane (TM) bundle is usually unwound [18,19,20,21,22,23,24,25,26,27,28]. The situation for ligands of rhodopsin-like ligands is diverse: Some peptides are bound with their N-termini towards the TM bundle, others with their C-termini. The ligands typically change their conformation upon binding, but this may occur in both directions: order to disorder or vice versa (e.g., [8,57,58,59,130]). 

Several concepts have tried to rationalize the initial receptor recognition and specificity, like the message-address [131] or membrane-compartment concepts [132,133,134]. The message-address concept introduced in the late 70s suggests that different epitopes contain the selectivity-determining and activity-determining residues. This concept works well for the opioid and adrenocorticotropic hormone systems. For most other peptide GPCRs, however, these epitopes overlap and cannot be unambiguously assigned. In contrast, the membrane-compartment concept appears to be applicable to many peptide ligands (for instance, [135,136,137]). It is suggested that the membrane-bound state of the peptide/protein ligand is a part of the binding trajectory, and constrains the different distributions of random coil conformations, and/or pre-orients a peptide for the respective binding pocket. 

The most powerful technique to investigate conformational changes occurring with the formation of the ligand–GPCR complex is nuclear magnetic resonance (NMR). It observes the chemical environment, and hence, the chemical shift of nuclei with an odd number of protons and neutrons, which creates a nuclear spin. NMR active nuclei include ^1^H, ^13^C, ^15^N and ^31^P. NMR can provide residue-resolved insights into the structure and dynamics of the peptide in the process of binding. Moreover, NMR may inform about interfaces/residues that contact the receptor, and help unraveling the recognition process. NMR can observe a set of residues simultaneously, and the high spectral resolution of multidimensional NMR experiments even permits the observation of the entire peptide.

NMR requires microgram-milligram amounts of pure peptide/protein ligand with NMR-active isotopes. For some applications, the naturally abundant ^1^H is sufficient and the experiments can be performed without any labeling. For multidimensional approaches, the ligand often needs to be labeled with ^15^N or ^13^C isotopes. Isotope-labeled peptides can be assembled straightforwardly by solid-phase peptide synthesis using commercially available building blocks and standard coupling procedures (e.g., [57,130,138]). In addition to the labeled peptide, NMR experiments require milligram amounts of the cognate receptor. The receptor must be available in a functional form in detergent micelles, lipid bicelles or other model membrane systems, and being reasonably stable in the required concentration, which is typically the experimental bottleneck (as reviewed [139,140,141]). Receptors can be purified from large-scale expression of intact protein in eukaryotic cells (yeast, insect cells, or mammalian) with or without introducing thermo-stabilizing point mutations or fusion proteins, very similar to common workflows in protein crystallography [140,141]. Alternatively, receptor expression in *E. coli* inner membranes or as inclusion bodies and subsequent in vitro folding can provide high amounts of functional receptor [139,141]. Moreover, this strategy allows for the straightforward and cost-efficient incorporation of isotopic labels into the receptor, as required for the investigation of the receptor structure and dynamics by NMR (see below). 

Depending on the affinity of the peptide–receptor interaction and the membrane system used to reconstitute the receptor, NMR experiments can be performed either in solution or solid state. In solution NMR, the molecules exhibit fast, isotropic motions in the solvent and the measured resonances report on the chemical environment and internal dynamics of the residues. However, large membrane protein complexes rotate slowly in the solvent, which leads to anisotropic samples. Signals obtained from these samples show broad line widths, which limits the application of solution NMR to GPCR complexes [142,143,144,145]. Moreover, the signals of the bound ligand appear broadened and eventually become undetectable when the ligand exchanges between bound and unbound states slowly, with rates of ~1 ms^−1^ (exchange broadening, reviewed in [146]), which is typical for high-affinity peptide ligands. 

In solid-state NMR, the analyte is either frozen or lyophilized which leads to a defined orientation. The samples are aligned in the magnetic field, and the magnitude and orientation of nuclear spin interactions (e.g., bond vectors) are recorded, rather than the isotropic values of chemicals shifts (reviewed in [147]). As a consequence, there are no size-limitations for solid-state NMR. Importantly, this technique still permits the investigation of internal protein dynamics. In the present manuscript, we will restrict ourselves to a few key principles and highlight applications of solution and solid-state NMR to study the dynamic peptide–GPCR complexes. We refer the reader to recent excellent reviews [148,149,150] for more detailed methodological descriptions and illustrations.

### 3.1. Solution State NMR for Low-Affinity Ligands

To To investigate the bioactive structure of receptor-bound peptide ligands featuring low to moderate binding affinity, solution state NMR is mostly applied. The experiments exploit the fact that the nuclear spins ‘memorize’ the receptor-bound conformation, while the peptides dissociate fast enough from the receptor so that the spin relaxation can be measured in the free state. The approach is only applicable to ligands featuring exchange rates (k_on_ + k_off_) faster than ~1 ms^−1^. Most widely applied are transferred nuclear Overhauser effect spectroscopy (trNOESY) measurements. The principle is essentially similar to the classic NOE-experiment [151,152], which measures dipolar coupling of two nuclei if they are closer than 5 Å (can be backbone or side chain atoms). In transferred NOEs, the carryover of the spin state from the bound to the unbound state leads to a change of sign of the NOE signal relative to the diagonal [153,154], which facilitates identifying the ligand signals, for instance in the uniformly labeled ^1^H-^1^H NOESY situation. From trNOESy experiments, a high number of short-range (<5 Å) intramolecular distances (structural restraints) can be derived, which permit an accurate calculation of the structural ensemble of the bound ligand. 

Using ^1^H-^1^H trNOESY, the receptor-bound structure of an engineered low-affinity variant of the pituitary adenylate cyclase activating polypeptide (PACAP^1-21^) [155] was determined. Structural studies for PACAP by NMR were only made possible by truncating the peptide sequence to significantly reduce the K_d_ from 3 nM to 18 μM, while the agonistic properties were retained [155]. Residue assignment was accomplished by using only ^1^H-^1^H trNOEs, which resulted in >350 structural restraints, including side chains. In the receptor-bound state, PACAP^1-21^ retains the C-terminal α-helix characteristic for ligands of the secretin family. The N-terminal segment, which binds deep into the transmembrane crevice to activate the receptor, adopts a unique conformation with residues 1-2 extended, followed by two consecutive β-turns (‘β-coil’) formed by residues 3–7 [155]. This finding is in apparent contrast with the very recent cryo-EM structure of PACAP^1-27^ bound to the PAC_1_R [24], which displays an extended α-helix up to the N-terminus. This, however, may also indicate the conformational flexibility of the peptide, which is not captured in the structural snapshot of cryo-EM. 

Such high conformational flexibility of the “activating” part of the peptide has been observed in other systems, such as a 13-mer peptide fragment of dynorphin bound to the κ-opioid receptor [156]. The peptide featured an affinity of about 200 nM and displayed fast k_on_ and k_off_ at the κ-opioid receptor, making this system ideally suited for solution NMR. The peptide was labeled (^13^C, ^15^N) at 9 out of 13 residues. Using trNOESY experiments, >50 structural restraints were determined and used for structural calculations. The peptide comprises one helical turn in the central part (L^5^-R^9^), while the N- and C-terminal part are flexibly disordered. The surprisingly dynamic nature of the N-terminal sequence, which comprises the activating “message” in the well-established message-address concept of the opioid receptors [157], was further confirmed by direct T2 relaxation measurements. Together, this indicates multiple bound states with various ensembles of receptors. 

Similar patterns of structured and less structured parts in the bioactive peptide N-terminus were also found for ghrelin binding to the GHSR_1a_ using the same experimental strategies [158]. Specifically, residues F^4^-L^5^ of ghrelin build up a very rigid hydrophobic core, together with the unique acyl moiety attached to S³ of the peptide, while the extreme N-terminus (G^1^, S^2^) remains more flexible, in line with an independent study using solid-state NMR [159]. 

Two further techniques of NMR allow identifying the residues of the peptide ligand that interact with the receptor. These are saturation transfer difference spectroscopy (STD, reviewed in [149,154]) and transferred cross saturation spectroscopy (TCS) [160]. The latter requires ^15^N/^13^C and ^2^H labeling of the ligand. Both techniques are based on the transfer of magnetization from receptor to the ligand. In either case, the receptor is irradiated at frequencies away from resonances of the ligand, but the magnetization is transferred to the ligand across the receptor–ligand binding interface. If the ligand features a sufficiently fast k_off_, the cross-saturated signals are detectable in the free state, and can be compared with the signals measured in the unbound state. STD has been applied on receptor-bound ghrelin [159], whereas TCS has revealed the interaction interface of CXCL12 (68 amino acids) at the CXCR_4_ [161], and MIP1α (69 amino acids) at the CCR_5_ [162].

### 3.2. Solid-State NMR of High-Affinity Ligands

The k_off_ of high affinity peptide ligands is not fast enough for determining their receptor-bound structure by solution NMR. In such cases, solid-state NMR can be applied. Typically, solid-state NMR yields a lower number of structural restraints compared to solution NMR, and provides information only on the backbone conformation, while the structure of the side chains or residue contacts arising from secondary or tertiary structure are not resolved. Importantly, while solution state NMR extensively measures resonances of ^1^H nuclei, solid-state NMR focuses on ^13^C and ^15^N nuclei. There is an empirical correlation between the spectral position of C_α_ and C_β_ resonances of an amino acid residue and its conformational angle ψ, which in turn correlates with the secondary structure of a polypeptide chain. A chemical shift index (secondary chemical shift) can be calculated, which is defined as Δδ = (C_α_ − C_β_)_observed_ − (C_α_ − C_β_)_random coil_. The random coil values are taken from high resolution NMR structures of soluble proteins and are available in reference literature and databanks [163,164,165]. Negative Δδ values are observed in β strand conformations (large positive value for ψ angle), while a positive Δδ reflects helical conformations with ψ < 0. The structural ensemble of the peptide chain can be calculated by ‘reverse’ or ‘forwards’ approaches. In the reverse approach, the backbone angles matching a calculated chemical shift index are extracted from high-resolution structural databases via the program TALOS on the basis of the chemical shift and sequence similarity [166,167]. The ‘forwards’ approach predicts instead a large ensemble of peptide conformations in silico, simulates the chemical shifts, and filters for the best matching solutions [57]. 

To discern the signals of ligand from those of the receptor and background signals, ^13^C labeling of the ligand is required. In addition, the signals of the ^13^C nuclei naturally occurring in the receptor and membrane-mimetic (present in large excess by mass) must be ‘substracted’ from the spectra. This is done by selecting pairs of directly bonded ^13^C nuclei (double quantum filtering techniques, 2QF), which is rarely met by naturally abundant ^13^C nuclei. A pioneering study has optimized 2QF sequences and recorded ^13^C-^13^C correlation spectra for a uniformly labeled truncated version of neurotensin (NTS^8-13^) bound to the NTS_1_R [130]. The corresponding backbone angles were then extracted in the ‘reverse’ approach. Compared to the unbound state [122,123,130], NTS^8-13^ transitioned from a disordered to a β-strand conformation. This conformation was later observed also in the crystal structure of the same complex [8]. A very similar approach was applied to determine the structure of the 9 amino acid peptide bradykinin bound to the B_2_R [138]. A transition of the C-terminus from a disordered state into a well-defined β-turn was observed, and overall the peptide displayed a S-like fold with a more structurally diverse N-terminus (Figure 1B).

Expanding this strategy to larger peptide ligands, we have reported the conformation of NPY_13-36_ bound to the Y_2_R [57], and that of NPY_1-36_ bound to the Y_1_R [58], which required in total 10/14 peptides with up to four ^15^N/^13^C labeled positions to reduce ^13^C signal overlap. The backbone conformation was calculated in the ‘forwards’ approach due to the length of the peptide [57]. NPY features in buffer [119] and when bound to lipid micelles [120] an amphipathic α-helical structure that extends from P^13^ to the C-terminal Y^36^. In the ligand-bound state, the very C-terminal residues unwind from the amphipathic α-helix starting at T^32^ at the Y_2_R [57], while at the Y_1_R, the α-helix is retained up to position R^33^ [58]. This suggests that fine-tuned C-terminal unwinding contributes to subtype selectivity in the NPY system. Further solution NMR experiments at the Y_2_R showed a significantly altered chemical environment (chemical shift) and slowed exchange between different states (observed as line broadening) in the region of the amphipathic helix. These experiments had to be carried out with sub-stoichiometric amounts of the receptor to avoid extreme line broadening of the signals due to the slow exchange kinetics (high-affinity ligand). Thus, the ligand resonances are a mixture of free and bound states, but nonetheless give a qualitative view on structural changes of the ligand. Guided by these data, double cycle mutagenesis identified extensive hydrophobic interactions between the β-strands in the ECL2 of the receptor and the central helix of NPY [57]. This finding has reinforced prior suggestions that NPY might be recognized from the membrane bound-state [136]. In terms of the binding trajectory, the ECL2 might initially take up the ligand from the membrane-bound state, retaining extensive hydrophobic contacts of the hydrophobic side of NPY’s helix, and position the C-terminus into the TM binding pocket where it is unfolded and accommodated into high-affinity polar interactions.

Recent improvements of NMR techniques have enhanced the sensitivity of the NMR measurements. This has made NMR applicable to the study of ligands bound to GPCRs obtained from expression platforms that give low yields. Dynamic nuclear polarization (DNP) combined with solid-state NMR enhances the detection sensitivity by about 100-fold compared to conventional NMR experiments. In DNP experiments, nuclear spins are not polarized directly, but via polarization transfer from stable electron radicals in the solvent irradiated by microwaves. The polarization transfer can be combined with diverse correlation experiments and pulse sequences [168,169,170]. Using this novel technique, the fold of desR^10^-kallidin (DAKD) when bound to the bradykinin receptor subtype 1 (B_1_R) was determined, using as little as 300 μg receptor per sample [59]. Kallidin is a 10-mer peptide that shows high homology to bradykinin and activates B_1_R and B_2_R with similar potency. Removing the C-terminal R^10^ of kallidin leads to almost exclusive binding of the resulting DAKD to the B_1_R [59]. DAKD shows a V-shaped geometry with a central β-turn between residues P^3^ and F^6^, which is observed both in the free and in the B_1_R-bound state. This conformation is strikingly different from the S-shaped fold observed in bradykinin bound to the B_2_R (see above), which instead underwent large structural transitions upon receptor binding [138]. Comparative models of B_1_R and B_2_R suggested that the two receptors distinguish DAKD and bradykinin on the basis of both chemical and conformational factors. On the one hand, about one third of the engaged sequence counterparts are non-conserved. On the other hand, the strikingly different backbone folds force the engagement of specific residues whose sequence counterparts are not engaged in the respective other receptor. The lack of major structural rearrangement of DAKD in solution compared to the B_1_R bound state is suggested to be related to the high basal activity of this receptor, assuming a conformational selection mechanism.

## 4. A Receptor’s Perspective

Unlike the structure of peptide ligands, the overall structure of GPCRs is well conserved, with most diversity seen in the N-and C-termini and loop regions. Despite this defined architecture and secondary structure, GPCRs are highly dynamic and feature multiple conformational states at different energy. Different states show different affinities for different ligands und effector proteins and take over different functions. Moreover, the current paradigm of GPCR activation by agonist binding involves a process called ‘allosteric coupling’, i.e., structural changes in the extracellular ligand binding pocket are conformationally linked to the intracellular G protein/arrestin-binding site. This is a reciprocal process: agonist binding leads to structural changes in the intracellular binding site, and effector-binding changes the conformation of the ligand binding pocket. Different ligands can induce different conformational changes, which are transmitted to the intracellular binding pocket, thus favoring the binding of one effector over another. This structural framework is the basis for the principle of “biased signaling”. While most GPCR agonists trigger several signaling pathways (e.g., both G protein and β-arrestin), biased ligands address only a subset of receptor conformations, thus activating only a subset of receptor functions (functional selectivity). Biased ligands hold great pharmacological potential since they may dissect therapeutic benefit from side effects [171,172]. 

The ensemble of the different conformational states accessible to a GPCR defines its energy landscape (Figure 1C). It is currently accepted that the conformational exchange between different activation states occurs in the ms timescale. Single amino acids act as microswitches, which change the pattern of their interactions, and/or their dynamics (exchange rate). These switches serve as connector between the binding pocket of the ligand at the extracellular side and the binding sites of the intracellular effectors. The process of receptor activation is apparently governed by conformational selection, with agonists distinctly stabilizing pre-existing, but sparsely populated conformations [173,174]. In addition to the rather slow global rearrangements mediated by microswitches, GPCRs permanently undergo side-chain repacking and segmental fluctuations [175] which occur on a fast (<μs) timescale. This leads to surprisingly large amplitude motions of > 30° along the protein backbone in all activation states [176,177,178]. This flexibility certainly contributes to the microswitch rearrangements, but might also have direct functional contributions in regions aside of the microswitches. The modulation of the fast fluctuations in different activity states and receptor loci is currently not clear, and has only been addressed by a few studies. 

**Figure 3:**
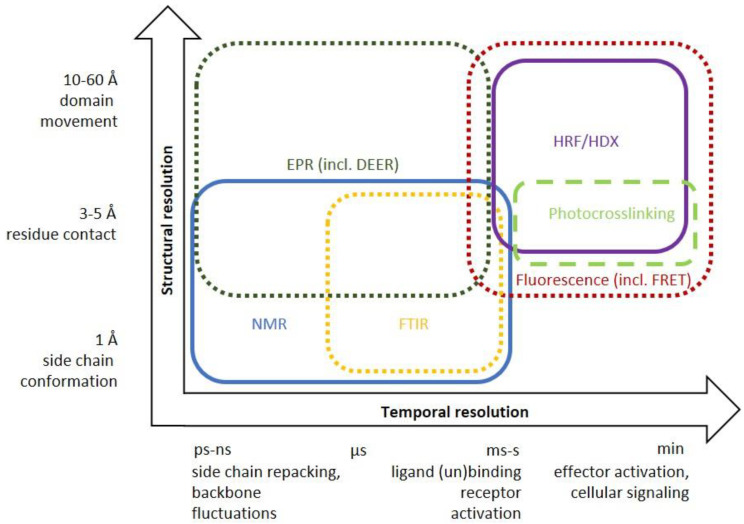
Structural and temporal resolution of methods to capture the structural dynamics of ligand–receptor interactions. The *y*-axis indicates the range of structural resolution, and the *x*-axis illustrates whether the method is sensitive to the fast and/or slow dynamics of the ligand and receptor. For instance, EPR-based methods (dark green box) can detect changes of residue contacts as well as domain movements, and are sensitive to fast backbone fluctuations, but also the slower interconversion of inactive to active states (confer Figure 1C). Methods that can inform about many sites/domains in a single experiment are enclosed in solid lines (NMR, HRF/HDX). Boxes with dotted lines indicate methods that entail site-specific labeling of 1 or 2 sites per experiment and provide more sparse structural information (EPR, fluorescence). Photocrosslinking typically involves one reactive residue per experiment that is free to react with the entire protein, and the experimental design often aims for large scale scanning mutagenesis (dashed line).

Transitions between receptor states can be monitored by spectroscopic techniques (Figure 1C), foremost NMR, which also allows calculating the number of distinct states, the transition rates and energy barriers along the activation pathway. Other biophysical techniques to illuminate the structural dynamics of GPCRs on different time- and structural scales include EPR, fluorescence, FTIR, and HDX/HRF coupled with mass spectrometry (Figure 3, Table 1). We will now briefly present these techniques, including aspects related to sample preparation and highlight findings for peptide/protein-activated GPCRs.

### 4.1. Selection and Specific Labeling of Sites-of-Interest

Each technique investigates the behavior of a specific biophysical probe, which may be already naturally present or must be installed onto the receptor. In any case, biophysical analysis of GPCRs with complete resolution of all residues and their specific properties will remain technically impossible. Thus, a set of ‘diagnostic’ positions that inform about the region(s) of interest needs to be selected. These residues are typically chosen by comparing different crystal structures, which have identified common allosteric switches/connectors that subtly change their conformation leading to large-scale rewiring of contacts and active receptor conformations [29,30,31,33,34,179,180,181]. The selection of sites-of-interest is certainly most straightforward if the atomic structure of the receptor has been determined, although homology models give workable starting points [182,183,184]. By combining information from several sites, a comprehensive picture can be constructed for a given receptor, which covers the energy profile and structural alterations of different loci when the receptor is stimulated with different ligands or interacts with different transducers.

Large proteins, including membrane proteins, can be labeled using a residue- or site-specific approach. For residue-specific labeling in NMR, it remains most straightforward to ‘simply’ add single ^13^C/^15^N labeled amino acids to the expression medium [185]. One potential complication is isotopic scrambling (i.e., ^13^C/^15^N labeling of non-selected amino acids) resulting from the metabolism of the expression host. In *E. coli*, this can be circumvented by auxotrophic strains [186], or the use of cell-free expression techniques (reviewed in [187]). Depending on the amino acid frequency in the target protein, some positions may be mutated to avoid crowded spectra. Also the assignment of the signals to a specific residue is typically done by mutagenesis (mutation of this residue removes its resonances from the spectrum). However, residue mutagenesis comes at the risk of altering the local environment or even the global structure, which can dramatically change the NMR spectra. As an alternative, residue-specific ^13^C signals can elegantly be assigned without mutagenesis through correlations to a successive ^15^N-labeled residue by NCOCX correlation spectra, provided each of the residues of interest is followed by a different amino acid, leading to unambiguous residue pairs [188].

Dyes for fluorescence spectroscopy, EPR, but also ^19^F-NMR labels are attached site-specifically to one or two positions. The labels are typically installed post-translationally by mild and chemoselective reactions (bioorthogonal chemistry). Most frequently, the canonical Cys residues, either endogenous or introduced by mutation, are targeted. Thanks to the uniquely high nucleophilic character of the thiol side chain, Cys can be selectively modified by methanethiosulfonates, maleimides, or iodacetamides carrying the label of interest [189,190]. To avoid spurious labeling, the receptor should be depleted of other cysteine residues, which may have functional consequences. Nonetheless, there are several successful examples of GPCR labeling at Cys residues, including rhodopsin [191], β_2_AR [192], GHSR_1a_ [193], Y_2_R [194], and AT_1_R [173]. Alternatively, labels can be introduced through chemical or enzymatic reactions at peptide tags, although this method is mostly limited to the N- terminus (reviewed in [195]). Techniques of protein ligation and semi-synthesis [196] allow tailored labeling schemes for biophysical investigations of GPCRs, as well as their ligands and effector proteins. For instance, native chemical ligation has been used to attach a phosphorylated, isotopically labeled C-terminus to the β_2_AR receptor, and investigate the mechanism of arrestin binding [197].

Modern techniques of genetic code expansion have opened up other possibilities for protein labeling, including GPCR labeling [77,198]. In particular, a series of chemical anchors have been genetically encoded that can be selectively labeled with desired probes using bioorthogonal chemistry [77]. Importantly, this approach does not require working in a Cys-free background. In the first examples of GPCR labeling on ncAA anchors, the same *p*-azido-Phe (Azi) that we have discussed as a photo-crosslinker was applied for either copper-catalyzed or catalyst-free (strain promoted) azide-alkyne cycloaddition to label rhodopsin in vitro (CuAAC and SPAAC, respectively) [199,200]. Other ncAAs bear biophysical probes suitable for studies of GPCR dynamic directly on the side chain, as for instance EPR probes [201,202,203]. In addition to being a crosslinker and a bioorthogonal anchor, Azi can be applied as a biophysical probe itself, as it gives a unique vibrational signature in infrared spectroscopy (IR) [204]. 

### 4.2. NMR: Investigation of Conformational Equilibria

The most powerful technique to investigate ligand-induced conformational transitions in GPCRs reconstituted in lipid environment is NMR spectroscopy, usually in solution state, but also in the solid state. Indeed, results of NMR studies have shaped our current understanding of GPCR activation and allosteric communication. NMR can provide information about how different ligands act on a receptor, i.e., what is the structural basis for their function, be it agonism, biased agonism etc. If, for instance, a ‘target region’ for G protein-biased agonism can be identified, this may allow the design of more specific drugs. 

NMR can observe simultaneously a pool of suitably labeled residues (in general up to ten) distributed along the receptor and report in a very sensitive fashion on changes of their chemical environment. In such experiments, the coexistence of multiple conformations on the ms timescale reflects in the splitting of residue resonances. NMR can also report on exchange rates by specialized experiments, for instance Carr-Purcell-Meiboom-Gill (CPMG) measurements, reviewed in [146]. Moreover, also conformational dynamics on a much faster timescale (<μs) can be addressed directly to resolve side chain fluctuations and backbone amplitudes [146] (Figure 2). To investigate conformational transitions of GPCR, a set of residues need to be labeled with ^13^C. A very popular approach consists in substituting all residues of one kind with the NMR probe (Table 1). Labeling is often performed at Met sites [205,206,207,208,209,210], as GPCRs usually carry only a few Met residues, typically well distributed across the sequence. Labeling is performed by supplementing the expression medium with **^13^_ε_CH_3_-methionine**, which does not alter the chemical features of the amino acid. Installing the ^13^C label on the terminal CH_3_ of the thioether allows profiting from the sensitivity and favorable relaxation properties of this flexible side chain in solution NMR because of the three-fold multiplicity of the proton signal and the fast rotation of the methyl group, which partly uncouples this group from the slow overall tumbling of the large GPCR complex [211,212].

The first NMR studies of GPCRs investigated the prototypical β_2_-adrenoceptor (β_2_AR). In this receptor, a Met (M^2.53^, GPCR residue numbering follows Ballesteros and Weinstein [213]) is located just below the binding pocket but without direct ligand contacts. It was shown that the chemical shift of this residue correlates with the efficacy of agonists [205,207]. In the inactive state, this residue yields two resonances in slow exchange (seconds), providing direct evidence for structural heterogeneity. Upon binding of a weak partial agonist, the transition between inactive and active-state, measured on the basis of the exchange rate of the M^2.53^ resonances, occurs in the millisecond time-scale [207]. Agonist stimulation also affected signals of M^5.54^ and M^6.41^ in TM5/6 towards ICL3 of β_2_AR [205,206]. By adding a G protein mimetic to the agonist-bound receptor, additional changes were observed in the chemical environment and population states of these two residues [206]. This was the first biophysical demonstration of the coupling between the ligand binding pocket and the G protein interaction interface, whereby the G protein (or a G protein mimetic) stabilizes a subset of active-like conformations [206]. This discovery has been supported by many biophysical and biochemical studies since (e.g., [174,208,209,210,214,215,216,217,218,219,220]). 

Strong structural heterogeneity has been observed also in peptide receptors. NMR studies at the μOR revealed allosteric coupling at this receptor, with conformational exchange in the low ms-timescale [208,209]. To address extra- and intra-cellular solvent-exposed regions, in one study labeling was performed at lysine residues, using **^13^C-dimethyl-lysine** [209]. This label is generated by in situ reductive methylation of the ε-amino group of native lysine residues. This yields a tertiary amine, which retains the positive charge, but features the sensitivity and favorable relaxation properties of methyl groups [221,222] similarly as ^13^_ε_CH_3_-methionine probes. Interestingly, agonist stimulation significantly altered the conformational state of the ECL2 [209], which was even more pronounced upon the co-binding of agonist and a G protein mimetic nanobody. All spectral changes appear very similarly when stimulated with the synthetic small molecule agonist BU72 compared to the (synthetic) peptide agonist Dmt^1^-DALDA, which suggests a common activation mechanism.

**^19^F NMR** is another powerful approach to detect conformational changes at GPCRs, which has been particularly applied to explore conformational changes at the intracellular side of GPCR and study the recognition of different effector protein in the frame of biased signaling [174,214,218,223,224,225,226,227,228,229,230]. As ^19^F is not naturally present in proteins, the method displays excellent sensitivity even in one-dimensional measurements, including van-der-Waals packing and electrostatic interactions [231,232]. ^19^F NMR probes are chemically attached to single Cys residues using labeled probes (e.g., 2,2,2-trifluoroethanethiol or 3-bromo-1,1,1-trifluoroacetone), which may affect the natural dynamics of the position. Complementary insights can be obtained by testing different attachment sites and different probes [225,229,231]. ^19^F labels installed at the ends of TM6 and TM7 of the β_2_AR revealed that arrestin-biased ligands predominantly affect the conformation of TM7, and G protein-biased ones that of TM6 [223], which provided for the first time a structural basis for biased signaling (Figure 1C). 

In addition, ^19^F NMR experiments allow extracting entropic and enthalpic parameters for each conformational state, which allows defining the energy landscape of receptor activation and shows how agonists change the prevalence of each state. Studies at the β_2_-AR suggest that receptor activation is enthalpically disfavored and entropically driven [225,229]. In other words, agonist binding favors receptor activation by inducing greater motional amplitudes of certain receptor regions, and possibly the release of highly ordered water molecules into the environment, and not by their binding enthalpy.

In addition to the presented examples with ^13^C-methionine, ^13^C-dimethyl-lysine, and ^19^F labels, other labeling schemes for NMR studies of GPCRs are possible: Isotopically labeled tryptophane may be used at endogenous positions [188,217,233,234] or as extrinsic probes [235]. Backbone assignments of ^15^N-labeled valine [236], or side chain assignments of ^1^H_δ1_,^13^C_δ1_-isoleucines [175] have also been applied to monitor the structural plasticity of individual receptors.

### 4.3. NMR: Contribution of Fast Side Chains and Segmental Dynamics?

There are also a few NMR studies that have addressed conformational dynamics of GPCRs on a much faster timescale (< μs), which reveal fast chain repacking and backbone fluctuations. Changes in the side chain dynamics have been shown to importantly contribute to entropy changes and thus free energy of binding of protein–ligand interactions in general [237,238]. Specific changes in the fast dynamics of receptors induced by different ligands, including biased agonists and allosteric modulators, might be correlated with the functional efficacy.

A study of the adenosine A_2A_ receptor demonstrated that different ligands differently modulate the fast side chain dynamics (ps-ns timescale) of ^13^C_δ1_ labeled isoleucine side chains [175]. For instance, both I^3.40^ in the conserved hydrophobic triad below the binding pocket and I^7.57^ at the intracellular tip of TM7 showed greater flexibility when the receptor was bound to an agonist compared to an inverse agonist. It is possible that the lower side chain flexibility at position I^7.57^ observed when bound to the inverse agonist contributes to hindering the effector recognition. In contrast, I^6.40^ close to the allosteric Na^+^ pocket was highly flexible irrespective of the ligand bound. Thus, the changes in the sidechain dynamics between the extracellular and intracellular domains appear to be loosely coupled. 

A second study measured fluctuations of the backbone of six ^13^C-labeled tryptophan residues in the neuropeptide Y_2_ receptor on a fast timescale (correlation times < 40 μs) [188]. In this case, although large motional amplitudes (~30–40°) of the Trp residues were observed, no significant changes of backbone mobility in the fast timescale were found between the ligand-free, agonist-bound, and arrestin-bound states of the receptor. On the other hand, the same study observed significant alterations in chemical shift and exchange dynamics on the slow (ms) timescale, which was attributed to localization of these Trp residues in microswitches, such as the W^6.48^ ‘toggle switch’ [188].

Other studies determined the overall dynamics of GPCRs on the fast timescale without site-resolution, including a few peptide/protein GPCRs [176,177,178]. The receptors were overall very mobile (motional amplitudes of the backbone > 30°), but no significant changes of the averaged mobility were detected in different activation states. Thus, it appears that changes in the local dynamics might be very specific to ligand, receptor, and particularly receptor-position. 

### 4.4. Electron Paramagnetic Resonance (EPR)

Electron paramagnetic resonance (EPR) can be regarded a ‘talented little brother’ of NMR that complements NMR at lower resolution and longer-ranged interactions (Figure 3, Table 1). EPR observes spin transitions of unpaired electrons induced by microwaves in the presence of an external magnetic field. Due to the higher frequency of electromagnetic radiation (microwave vs. radio frequency), EPR is about 1000-fold more sensitive than NMR, and EPR samples can be measured in the μM concentration range.

Electron spins do not naturally occur in GPCRs or their ligands, and need to be installed site-specifically at one or two positions per experiment. Typically, a sterically shielded nitroxide radical (NO•) in a tetramethylpyrroline ring is attached to a cysteine residue via a suitable linker [239,240]. The prototype of these labels is MTSL [241], which allows specific and reversible labeling of cysteine residues, only perturbs minimally the protein structure, and is well characterized regarding internal flexibility [240]. Alternatively, non-canonical amino acids carrying EPR probes can be directly incorporated using the expanded genetic code technology [202,203]. 

As originally conceived, protein EPR in a continuous wave setting (cw EPR) observes a single introduced nitroxide radical (NO•) at room temperature. This gives information about the chemical environment, solvent accessibility and the local dynamics in the fast timescale of 100 ps–1 μs within measuring times of a few minutes (reviewed in [240,242]). This is done by analyzing the lineshape and the observation of periodical spectral changes through collision with paramagnetic fast relaxing agents (O_2_—membrane compartment, Ni(II)ethylenediamine-diacetic acid—water soluble). The method is very informative: already 25 years ago, cw EPR experiments on spin labeled rhodopsin provided the first evidence that the mobility and the chemical environment of cytoplasmic regions of the receptor change upon activation [191,243,244]. Recently, cw EPR demonstrated a helical periodicity for the cytoplasmic helix 8 of the NTS_1_R, and a modestly increased mobility of most labeled sites upon agonist stimulation [245].

Signals of cw EPR become broadened in the presence of another nearby paramagnetic center. This is similar to NMR (spins are coupled and “sense” each other), however, dipolar coupling between two electron spins occurs over larger distances. This effect can be exploited to deconvolute distance information in the 8–25 Å range [242,246]. This range can be even more extended by pulsed EPR techniques termed DEER or PELDOR (double electron-electron resonance/pulsed electron double resonance), which enable distance measurements in the range of 8–70 Å [189,247], and thus nicely complements the short-range distance restraints that can be obtained from NOE-type NMR measurements. Pulsed EPR techniques are conducted in cryogenic conditions at low temperatures (50–80 K). The measurements yield a probability function, which can be mathematically deconvoluted into specific sub-states and population. In flexible systems, more than one distance peak is observed for a given pair of labeled residues, which reveals the existence of multiple conformational states, and thus also reflects the conformational dynamics on the ms-s timescale. For a global view on protein structure and conformational transitions, a number of pairwise distances needs to be recorded. 

Pulsed EPR (DEER) was first applied to rhodopsin [248,249,250] and has revealed the pattern of helix movements that accompany receptor activation, which include a 5 Å outward movement of TM6 and smaller changes for TM1 and TM7, while TM3 does not move [249]. In contrast to most other GPCRs, rhodopsin displays a stronger allosteric connection between the ligand binding pocket and its intracellular interaction interface, and follows a stricter sequential order of activation events [36,251], which likely originates from its specialized function. Nonetheless, a recent DEER-based study [248] has demonstrated that rhodopsin features different structural states in equilibrium. This conformational flexibility may contribute to the recognition of different cellular partners, i.e., transducin and visual arrestin.

More recently, DEER studies at the β_2_AR [214] and in particular at the angiotensin receptor (AT_1_R) [173] have revealed ligand-specific conformational changes in the receptor (Figure 1C). Using ten DEER pairs at the AT_1_R, a high conformational flexibility of the receptor was observed in the ligand-free state. Binding of the natural peptide agonist angiotensin, which fully activates Gq and arrestin-pathways (balanced agonist), further broadened the conformational distribution. This indicates that the angiotensin-bound receptor samples multiple of the pre-existing conformations and populates these states to a different extent, which is consistent with a conformational selection mechanism. In contrast, functionally selective (biased) peptide ligands stabilized different subsets of conformations, which were characterized by different relative distances of the TM helices and were rationalized into four structural patterns. Agonists that trigger G protein signaling stabilized a structure with a more open intracellular crevice, which *also* allowed arrestin binding. Instead, ligands that favor arrestin signaling stabilized more occluded conformations that do *not* permit G protein binding/activation. Moreover, differences were observed among the occluded conformations, suggesting that there are multiple structural patterns underlying arrestin-bias, possibly linked to different physiological consequences. The recently solved crystal structures of the AT_1_R bound to angiotensin and the same biased agonists [14] complement the findings of the EPR studies. While the crystal structures cannot recapitulate the complex conformational ensembles seen by EPR, it provided high-resolution clues on *how* the different ligands favor different conformations. The overall structural differences were surprisingly small, and only occurred in the deep transmembrane binding pocket around the modified C-terminus of the peptides. The F^8^ side chain of the native balanced peptide agonist displayed a high flexibility. This leads to an on-axis rotation of TM3, which releases the interaction of N^3.35^ with N^7.46^, rearranges hydrogen bond networks involving the NPxxY motif, and permits a fully active state with respect to TM7/H8. In contrast, arrestin-biased ligands did not induce TM3 rotation and were structurally more defined. Thus, the rotational freedom of the terminal F^8^ side chain seems to orchestrate intracellular conformations, which was confirmed by molecular dynamics simulations [252]. 

Finally, EPR can be applied to protein complexes. EPR studies revealed structural rearrangement in G proteins or their fragments when binding to GPCRs [253,254,255,256], as well as structural changes of activated arrestins [257,258], and even allowed mapping distances in the rhodopsin-arrestin complex [107,259].

### 4.5. Fluorescence

NMR and EPR are very powerful biophysical techniques to explore the energy landscape of GPCRs in vitro under equilibrium conditions. However, they require high amounts of pure sample. Fluorescence-based techniques offer an alternative approach to study structural dynamics of receptors at low nanomolar sample concentrations. Importantly, fluorescence experiments can be carried out in the natural environment of living cells, which is essential to validate and complement findings from spectroscopic techniques only applicable in vitro. Many studies have applied fluorescent techniques in vitro and in vivo, and it is beyond the scope of the present review to cover them all. Thus, we will focus only on a few examples and refer to more specialized literature where applicable.

Fluorescence spectroscopy with single labels at TM3 or TM6 has been applied to monitor the conformational changes of isolated GPCRs in the early 2000s, before large GPCR amounts have become available for NMR studies. Single environmentally sensitive fluorescent probes report on changes of the polarity or the pH around the fluorophore (Figure 3, Table 1). This principle has been used to monitor the activation and activation kinetics of the β_2_AR by different agonists [192,260,261,262,263]. For instance, a decrease of fluorescence intensity of a bromo-bimane installed at Cys^271(6.33)^ of the β_2_AR reflects the outward movement of TM6 related to receptor activation [264]. Other applications made use of fluorescence anisotropy to deduce the lifetime of certain (sub)-states in vitro [193,265,266,267]. Installation of two fluorophores with overlapping spectral properties at two positions enables distance determinations by fluorescence quenching [260,264] or Förster resonance energy transfer (FRET) [268]. FRET theoretically gives access to distances in the order of 20–60 Å (reviewed in [269]). A modified setting employing lanthanoids as long-lived donor species (LRET) [270,271,272] overcomes some practical problems of FRET, i.e., short half-lives of the fluorophores, and dependence on orientational factors which are hardly controllable and compromise accuracy of determined distances [269]. 

Moreover, FRET- and bioluminescence equivalent BRET-sensors have been used in living cells to look into conformational changes and their kinetics, and study the transducer interaction profile of receptors even without amplifying steps (e.g., [273,274,275,276,277]). This typically involves at least one fluorescence protein (or luciferase) that is genetically fused into internal loops or termini of GPCRs. The second fluorescent partner can be another fluorescent (or luminescent) protein that is installed in the same receptor (*intra*molecular application) or at an interacting protein (*inter*molecular application). Alternatively, smaller peptide tags (e.g., FlASH-tag) can be used to specifically introduce a small-molecule fluorophore into loops of GPCRs. In this regard, FRET/BRET is usually exploited as a primary readout of interaction/activation, and kinetic parameters can be resolved down to milliseconds. Intriguingly, the activation of different GPCRs, such as β_2_AR or muscarinic receptors in living cells occurred faster than 100 ms. This timescale of receptor activation rates agrees well with recent NMR studies of GPCRs with small diffusible ligands that used near-native lipid or micelle reconstitution systems [174,207,214,218,228], although the precise choice of detergent/membrane mimetic has a moderate influence on the activation rates of a given system [207,224]. Interestingly, the activation rate of the parathyroid hormone receptor PTH measured by intramolecular FRET in vivo was significantly slower with an activation rate constant of ~1 s^−1^. This rate constant coincides with the slower component in the two-step peptide binding process [278]. This creates the possibility that GPCRs activated by peptides might have a slower activation kinetics, which is limited by the binding trajectory of the ligand.

In addition, FRET/BRET sensors have qualitatively shown the existence of different structural receptor states and functional bias within the same system (e.g., [279,280,281]). This is revealed by different changes of the FRET/BRET efficiency in response to functionally selective ligands. However, more quantitative orientation and distance information is difficult to extract from FRET/BRET measurements with large fusion proteins, as the potential structural perturbation of these large probes along with long and flexible linkers hamper direct deconvolution of the information back to into the structure. 

Extending the advantages of fluorescence techniques regarding sensitivity and temporal resolution, brighter fluorophores and super-resolution microscopy have made possible single molecule fluorescence (smF) studies at GPCRs, both, in vitro and in live cells. This emerging method has been reviewed recently in great detail elsewhere [199,282,283,284], and we will thus restrict ourselves to a few key aspects. smF enables monitoring of the (de-)activation process of a single receptor at with structural (site-specifically labeled, e.g., change in FRET) and kinetic resolution (Figure 1C). This complements the information gained from structural and spectroscopic studies at equilibrium, and may visualize transient states that are visited only en route the activation pathway and, hence, not (detectably) populated under equilibrium conditions, as well as directly determine the rate of structural transitions slower than milliseconds. Moreover, the FRET spectrum can be deconvoluted into different structural sub-states [285], and thus, reports on the conformational equilibrium similar to the distance distributions obtained from pulsed EPR techniques (Figure 3). Impressive applications have been reported for the metabotropic glutamate receptor [286,287,288,289] and the β_2_AR [220,290,291,292,293]. In the latter case, the β_2_AR was labeled with a single, environmentally sensitive fluorophore at TM6 [292] or TM7 in vitro [293], specifically surface immobilized, and imaged by total internal reflection (TIRF) microscopy with 100 ms time resolution. Structural transitions in the ms-seconds time regime can then be directly observed as dwell times in the high- and low-fluorescent state, respectively. These measurements suggest that ligands modulate the kinetics of receptor conformational exchange, which can be specific to a certain structural site: The balanced agonist formoterol increases the frequency of activation transitions at TM6 [292], while the dwell-time of an active conformation of TM7 is more efficiently increased by an arrestin-biased agonist [293]. Another study at the β_2_AR [220] used double labeling at TM6 and TM4 with an optimized, environmentally *in*sensitive Cy3B*/Cy7B* FRET pair. Depending on the presence of agonists with different efficacies, the rate of structural transitions (observed as FRET differences) was measured. Interestingly, this revealed for the first time the persistence of high-FRET GDP- and GTP-bound β_2_AR-Gs complexes, in addition to the low-FRET complexes observed in the nucleotide-free state, suggesting that several intermediates are involved in G protein coupling [220].

To date, similar smF studies have not been reported for peptide-activated GPCRs yet. However, these techniques could be applied to study the binding trajectory of peptide/protein ligands to their cognate (and noncognate) receptors in the future.

### 4.6. Fourier-Transform Infrared Spectroscopy (FTIR)

A less well-known technique for the analysis of protein conformations and protein–protein interactions is Fourier-transform infrared spectroscopy (FTIR). FTIR is a technique relying on differential infrared light absorption (owing to vibrational resonance) of chemical bonds dependent on their electrostatic environment (reviewed in [294]). Technically, all absorption bands in the infrared region are recorded together as interferogram. Fourier transformation then deconvolutes the raw data to the actual absorption spectrum, which minimizes the measuring time and maximizes the signal-to-noise ratio, and makes it possible to measure μg quantities of protein. All polar bonds of the sample (protein) contribute to the IR spectrum, which leads to many overlapping bands. Similar to NMR, there are several characteristic regions in the spectrum. The amide I band (1700−1600 cm^−1^), reports on C=O stretch vibrations and is the most informative for protein structure changes. While the chemical structure of a protein cannot be deduced from the infrared spectrum because of band overlap, changes in the chemical structure can be detected by plotting the difference spectrum of two (or more) functional states. 

Thanks to the short measuring time, FTIR can detect activation intermediates with lifetimes as little as 1 µs (Figure 3, Table 1). Since this technique is not sensitive enough to detect single molecules, the protein samples need to be synchronized, for instance by light-induced activation (rhodopsin); or by ligand stimulation using stopped-flow instruments (~1 ms mixing time). Otherwise, the FTIR difference spectrum reports the weighted mean of the equilibrium condition.

FTIR has been widely used to address the activation process of rhodopsin [35,204,295,296,297,298,299,300,301,302,303], mostly taking advantage of the sensitivity of C=O vibrations to the protonation state of aspartate and glutamate residues and conformational changes [295,298]. For instance, FTIR in combination with UV–VIS has been used to develop a thermodynamic model of rhodopsin activation including the short-lived intermediates [298] and to investigate changes occurring in the intracellular side of the receptor upon binding of the Gα C-terminal peptide [35]. 

Proteins can also be equipped with IR-probes that resonate in spectral regions free of C=O or other vibrational background, such as azido or cyano groups [204,300]. These groups are incorporated site-specifically either by genetic code expansion [204,300] or using site-specific reactions, and are excellent reporters for changes of the local electrostatics. Genetic incorporation of *p*-azido-phenylalanine into four sites of rhodopsin along TM2, TM5 and TM6 (one at a time) revealed early conformational changes in the receptor, which occur before the formation of the active state [204,300].

### 4.7. Hydrogen-Deuterium Exchange (HDX) and Hydroxyl Radical Footprinting (HRF)

Moving away from spectroscopy-based techniques, mass spectrometry-based methods can reveal movements of protein domains in a timescale of seconds to minutes (Figure 3, Table 1). Hydrogen-deuterium exchange (HDX) or hydroxyl radical footprinting (HRF, also termed radiolytic footprinting) coupled with mass spectrometry detects structural transitions after a defined ‘stimulation pulse’. HDX detects the reversible exchange of backbone protons to deuterium upon exposure to solvent D_2_O (seconds-minutes). The exchange rate depends on the engagement of the proton in secondary structure elements, i.e., the amides in secondary structure elements are labeled more slowly (reviewed in [304]). HRF is characterized by an irreversible addition of highly reactive hydroxyl radicals (derived from radiolysis) to protein sidechains within seconds, and is not per se governed by secondary structures (reviewed in [304]). Both techniques typically require only micromolar protein concentration of receptor, without modifications, and give insights into the (change of) accessibility of certain receptor domains. This provides an excellent overview of the flexibility and accessibility of the entire protein in different states, with a sequence coverage of up to 80% for GPCR (complexes) under optimized conditions [41,305,306,307,308].

HDX or HRF can be applied to monitor ligand binding pockets over time [304]. For instance, differential HDX revealed distinct patterns of reactivity of the β_2_AR bound to agonists and inverse agonists [307,308]. Compared to the unbound receptor, inverse agonists stabilized both extracellular and intracellular regions whereas only the full agonist lead to higher accessibility (=destabilization) of cytoplasmic regions [307,308]. In agreement with NMR/EPR spectroscopic studies outlined above, different effects were observed with functionally biased compounds [308]. More recently, pulsed HDX and HRF was used to examine transient complexes forming during the process of G protein recognition at the β_2_AR and the A_2A_R [41]. The identification of transient complexes during the G protein–GPCR recognition that may serve as selectivity filter aligns well with complementary smFRET studies [220] (see above), as well as molecular dynamics simulations and crystallographic data [42], presenting a textbook example for the power of complementary biophysical studies to understand GPCRs.

## 5. Conclusions and Perspective

In summary, recognition of peptide ligands by GPCRs is a highly individual process that is hardly generizable into universal structural patterns. Mutagenesis and crosslinking techniques allow investigating structural and functional details of peptide–receptor interactions and provide unique information that is not accessible by mere structural investigation (crystallography, cryo-EM). Moreover, such biochemical methods target the receptor in the environment of the live cell, which is a complement to methods applicable only on isolated receptors in vitro. 

NMR studies have allowed investigating the structure of peptide ligands bound to their receptors. Many of the presented studies reveal distinct structural changes during ligand binding to GPCRs, and a surprisingly high dynamics of the peptide–receptor complexes. Investigation of multiligand/multireceptor systems like the kinin (kallidin/bradykinin) or the NPY family have shown that the activity determining residues adopt different conformations, change their conformation to a different extent compared to the aqueous state, and may form different contacts in the binding pockets. It remains poorly understood how the high specificity that characterizes peptide–receptors recognition is achieved. The structural changes of the peptide/protein ligands may follow the induced fit principle or occur as a consequence of conformational selection. In the latter case, the population of specific conformations may be too sparsely populated to be detected in the existing studies. 

It is conceivable that selectivity is at least partly encoded in dynamic processes and in the early binding trajectory, for instance by coarse charge-complementarity or extensive hydrophobic interaction surfaces that mimic membrane interactions. Thus, the access to the binding pocket can be prepared by events that pre-orient the ligand and involve receptor regions that are not part of the ‘final’ high-affinity binding pocket [309]. The dynamics of binding and the initial binding trajectory are extremely difficult, if at all, addressable by traditional experimental techniques. In the case of small molecule ligands, molecular dynamics simulations can provide insights into the process of recognition and binding [310,311], whereas peptide ligands are too large and complex for this kind of in silico predictions at the present time. The largest energy barrier for small molecule ligands during binding is suggested to be the first contact with the receptor, far away from the orthosteric binding position, likely because of the desolvation energy [309]. It is conceivable that a fraction of this energy barrier might be relieved by prior membrane-association of peptide ligands. Some of the intermediate binding positions were also assigned to biological functions [312,313,314]. Moreover, the presence of intracellular binding partners further modulates the thermodynamic profile and binding path [313,315,316]. Thus, there is a lot more to discover in the lively interactions between peptide ligands and cognate GPCRs.

NMR, EPR, FTIR and fluorescence-based methods have allowed investigating conformational changes occurring in the receptor upon ligand binding. GPCRs display high internal dynamics in the fast μs scale, and consequently large amplitude motions along the protein backbone in all activation states. Well defined microswitches change their interaction pattern, and/or their dynamics (i.e., rate of exchange between states) on a ms timescale, and serve as allosteric connector between the ligand and effector binding pockets to mediate the cellular responses. Several conformational states exist already in the apo state, and different subsets are stabilized by biased ligands [173], setting the structural framework for functional selectivity. On the basis of the present spectroscopic data, the receptor activation process seems to be governed by conformational selection, with agonists distinctly stabilizing pre-existing, but sparsely populated conformations [173,174]. As hypothesized for the ligand binding trajectory, also the prototypic signaling proteins are engaged sequentially, involving several intermediates [41,42,317,318]. All of these states can in principle be targeted pharmaceutically—once we have gained enough insights to address them specifically.

## Figures and Tables

**Table 1:** Biophysical methods to investigate structural dynamics of G protein-coupled receptors (GPCRs).

Method	Label	Introduced by	Provides Information About	Comments	Ref.
**NMR** (solution and solid-state)	^13^C_ε_-methionine or other specifically labeled aa: ^13^C/^15^N-tryptophane ^13^C_δ1_ isoleucine ^15^N-valine ^13^C_ζ_-tyrosine; ^13^C_β_-cysteine etc.	residue-specific during biosynthesis (expression medium)	chemical environment/distinct conformational states, exchange rates between different states can be extracted short-range interactions (via cross-polarization in solid-state NMR)	^13^C_ε_-methionine exploits favorable relaxation profile and flexibility of methyl-groups; deuterated background possible; ^15^N-valine: backbone assignment	[205,206,207,208,209,210] [217,233,234] [175] [236] [319,320]
	^13^C-(di)methyl-lysine	residue-specific; chemically: reductive methylation of primary amines with ^13^CH_2_O	chemical environment/distinct conformational states short-range interactions (participation in salt bridges)	exploits favorable relaxation profile and flexibility of methyl-groups; negative charge of side chain preserved	[209,321]
	^19^F	site-specific (1 label) chemically: (single) reactive cysteine plus TET or BTFA	chemical environment/distinct conformational states exchange rates between different states can be extracted short-range interactions (^19^F-NOE; ^19^F-^31^P REDOR)	direct excitation and no background allow straightforward extraction of entropic and enthalpic parameters <8 Å; up to 12 Å; e.g., phosphorylation sites	[174,214,218,223,224,225,226,227,228,229,230] [226] [322]
**EPR** continuous wave pulsed (DEER)	N-O· (nitroxide radical, typically stabilized by proximal dimethyl groups in a pyrrole ring)	site-specific (1 or 2 labels) chemically: reactive cysteines plus MTSL etc. Uaa labeling possible [201,202,203]	chemical environment, dynamics, accessibility (continuous wave) long-range distances (continuous wave, pulsed (DEER))	nitroxide scanning (SDSL) allows mapping of secondary structure and membrane boundaries, as well as structural transitions spectral broadening in continuous wave EPR 8–25 Å, DEER 20–70 Å	[191,243,244,245,323,324,325] [326,327,328] [173,214,249]
**Fluorescence Spectroscopy** FRET/LRET single molecule microscopy/spectroscopy	fluorophores	site-specific (1 or 2 labels) chemically: (single) reactive cysteines bioorthogonal ligation possible, e.g., “click”-chemistry at genetically engineered Azi; FlAsH-tags etc. [198,273]	shift of environment polarity lifetime of (sub)states (fluorescence anisotropy and lifetime) distances (quenching, FRET, LRET) rate of structural transitions (slower than ms) visualization of transient states not significantly populated in equilibrium	nM protein concentration sufficient, applications in living cells possible distance ranges: tryptophane-induced quenching 5–15 Å 20–80 Å with high orientational dependence for FRET, which is overcome by LRET sm fluorescence with environmentally sensitive fluorophores or smFRET	[192,260,261,262] [193,265,266,267] [260,264] [268] [270] [290,291,292,293] [220]
**FTIR**	intrinsic C=O bond vibrations N_3_ (or CN etc.) bond vibrations	label-free by genetic engineering, e.g., *p*-azido-phenylalanine (Azi)	protonation switches, secondary structure chemical environment (electrostatics)/distinct conformational states	application of difference spectra to extract responsive C=O signals kinetic resolution up to 1 μs bond vibrations in a region free of endogenous signals	[35,295,296,297,298,299] [204,300]
**HDX** **HRF**	(label-free)	in situ exchange proton-deuterium (reversible) in situ reaction hydroxyl-radical (irreversible)	accessibility, secondary structure, temporal resolution up to seconds accessibility, temporal resolution up to ms	Label free, global view on structural alterations Accuracy depends on coverage and resolution of mass spectrometry	[41,305,306,307,308] [41]

Abbreviations: Azi, *p*-azido-phenylalanine; BTFA, 3-bromo-1,1,1-trifluoroacetone; DEER, double electron-electron resonance; EPR, electron paramagnetic resonance; FlAsH, fluorescein arsenical hairpin binder; FRET, fluorescence/Förster resonance energy transfer; FTIR, Fourier-transform infrared spectroscopy; LRET, lanthanoid resonance energy transfer; MTSL, (1-oxyl-2,2,5,5-tetramethyl-pyrroline-3-methyl) methanethiosulfonate; NMR, nuclear magnetic resonance; NOE, nuclear Overhauser effect; REDOR, rotational-echo double-resonance; SDSL, site-directed spin labeling; TET, 2,2,2-trifluoro-ethanethiol; Uaa, unnatural amino acid.

## Abbreviations

A	active
A_2A_R	adenosine A_2A_ receptor
APJR	apelin receptor
AT_1_R	angiotensin receptor 1
Azi	*p*-azido-phenylalanine
β_2_AR	β_2_ adrenergic receptor
B_1/2_R	bradykinin receptor 1/2
BNPS-skatole	3-bromo-3-methyl-2-(2-nitrophenylthio)-3H-indole
Bpa	*p*-benzoyl-phenylalanine
BRET	bioluminescence resonance energy transfer
BTFA	3-bromo-1,1,1-trifluoroacetone
CCR_5_	CC chemokine receptor 5
CGRP	calcitonin gene-related peptide
CRF	corticopin-releasing factor
CRF_1/2_R	corticopin-releasing factor receptor 1/2
CuAAC	copper-catalyzed azide-alkyne cycloaddition
CXCL12	CXC chemokine ligand 12
CXCR4	CXC chemokine receptor 4
CPMG	Carr-Purcell-Meiboom-Gill (pulse sequence)
cwEPR	continuous wave EPR
DDM	n-dodecyl-β-d-maltopyranoside
DEER	double electron-electron resonance
ECD	extracellular domain
ECL	extracellular loop
EM	electron microscopy
EPR	electron paramagnetic resonance
ET_B_	endothelin B receptor
FlAsH	fluorescein arsenical hairpin binder
FRET	fluorescence/Förster resonance energy transfer
FTIR	Fourier-transform infrared spectroscopy
GLP-1	glucagon-like peptide-1
GLP_1_R	glucagon-like peptide-1 receptor
GLR	glucagon receptor
GPCR	G protein-coupled receptor
HDX	hydrogen-deuterium exchange
HRF	hydroxyl radical footprinting
I	inactive
LRET	lanthanoid resonance energy transfer
MIP1α	macrophage inflammatory protein 1α (=CCL3)
MS	mass spectrometry
MTSL	(1-oxyl-2,2,5,5-tetramethyl-*pyrroline*-3-methyl) methanethiosulfonate
ncAA	non-canonical amino acid
NMR	nuclear magnetic resonance
NOE	nuclear Overhauser effect
NPY	neuropeptide Y
NTS_1_R	neurotensin receptor 1
PACAP	pituitary adenylate cyclase-activating peptide
PAC_1_R	pituitary adenylate cyclase-activating peptide receptor 1
PTH_1_R	parathyroid hormone receptor 1
REDOR	rotational-echo double-resonance
SAR	structure activity relationship studies
SDS-PAGE	sodium dodecyl sulfate–polyacrylamide gel electrophoresis
SDSL	site-directed spin labeling
SPAAC	strain-promoted azide-alkyne cycloaddition
STD	saturation transfer difference spectroscopy
TCS	transferred cross saturation spectroscopy
TET	2,2,2-trifluoro-ethanethiol
TM	transmembrane
trNOESY	transferred nuclear Overhauser effect spectroscopy
Uaa	unnatural amino acid
Ucn	Urocortin
Y_1/2_R	neuropeptide Y receptor 1/2
